# Corrosion Monitoring in Petroleum Installations—Practical Analysis of the Methods

**DOI:** 10.3390/ma17112663

**Published:** 2024-06-01

**Authors:** Juliusz Orlikowski, Agata Jażdżewska, Iwona Łuksa, Michał Szociński, Kazimierz Darowicki

**Affiliations:** 1 Department of Electrochemistry, Corrosion and Materials Engineering, Chemical Faculty, Gdansk University of Technology, G. Narutowicza Str. 11/12, 80-233 Gdansk, Poland; juliusz.orlikowski@pg.edu.pl (J.O.); kazimierz.darowicki@pg.edu.pl (K.D.); 2 Rafineria Gdanska Sp. z o.o., Elblaska Str. 135, 80-718 Gdansk, Poland; a.a.jazdzewska@gmail.com; 3 Orlen S.A., Chemikow Str. 7, 09-411 Plock, Poland; iwona.luksa@orlen.pl

**Keywords:** corrosion, petroleum industry, corrosion monitoring

## Abstract

This paper presents the most typical corrosion mechanisms occurring in the petroleum industry. The methods of corrosion monitoring are described for particular corrosion mechanisms. The field and scope of the application of given corrosion-monitoring methods are provided in detail. The main advantages and disadvantages of particular methods are highlighted. Measurement difficulties and obstacles are identified and widely discussed based on actual results. Presented information will allow the corrosion personnel in refineries to extract more reliable data from corrosion-monitoring systems.

## 1. Introduction

According to the World Bank, the GDP in 2021 amounted to ca. USD 96.1 billion, out of which about USD 6 billion correspond to corrosion-related costs. Corrosion costs in the petroleum and petrochemical industry constitute a serious contribution to global losses. It is estimated that implementation of current best practices would help to avoid between 14 and 33% of the corrosion costs in the petroleum–petrochemical industry [1]. However, it is very difficult, if not impossible, to evaluate the losses related to environmental pollution, disability, or death of employees indirectly connected with corrosion problems. To limit the aforementioned hazards, several corrosion-monitoring methods and systems are implemented including the coupon method, the electrical resistance (ER) technique, the linear polarization resistance (LPR) technique, ultrasonic thickness measurement (UTM), or profile radiographic tests. A decision about the application of a particular method of corrosion risk measurement should be taken based on a detailed analysis of the technological process, parameters of the technological environment, design of installations and equipment, as well as rate and type of corrosion process.

**Figure 1:**
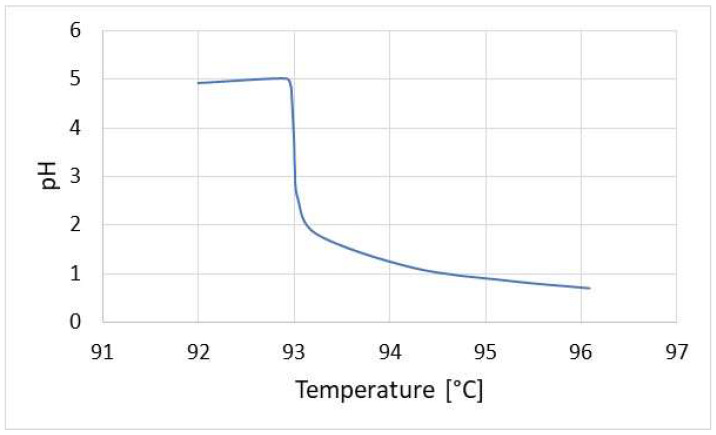
Dependence between pH of an aqueous phase and temperature of vapor stream from the atmospheric distillation column. The data were obtained from simulations with the Aspen Hysys 14.0 software.

The highest corrosion risk in refineries is connected with the installations where strong inorganic acids occur. An aggressive environment is most frequently gas phase, liquid phase, or a mixture of both. In some cases, hydrochloric acid, which is very well soluble in condensing water, is present in vapors inside crude oil atmospheric distillation columns. Braden et al. [2] and Duggan et al. [3] report that hydrogen chloride present in vapors inside the column originates in thermal degradation of the inorganic salts present in crude oil. Due to the high solubility of hydrogen chloride, the pH of the aqueous phase formed (onset of condensation) is very low, which results in dynamic local corrosion in a relatively narrow zone of water-condensation initiation. A small decrease in temperature causes the dissolution of ammonia and amines (added as the neutralizing agents), the effect of which is a negligibly lower corrosion rate. Figure 1 illustrates the aforementioned situation.

Due to the specificity of different solubility of gases, it is very difficult to prevent that type of corrosion. In a higher temperature range, under-deposit corrosion in ammonia chloride can occur [4]. Ammonia chloride is a salt of strong acid and weak base, which undergoes hydrolysis upon the presence of water vapor according to the following reaction:

NH_4_Cl(aq) = NH_3_(aq) + HCl(aq) (1)

The corrosion process takes a local form under the deposits of ammonia chloride formed. Corrosion monitoring of such streams is effectively conducted with coupon and ultrasonic methods due to significant corrosion losses. The deficit of the aqueous phase makes corrosion monitoring with the electrochemical methods possible only after the formation of a sour aqueous phase, which is disadvantageous due to its substantially lower corrosion aggressiveness compared to the vapor stream.

Another corrosion risk that calls for monitoring is corrosion in hydrofluoric acid. It concerns mainly HF alkylation installations. Pure hydrofluoric acid is not a corrosive medium. Upon contact of hydrofluoric acid with carbon steel, a stable layer of iron fluoride forms on the metal’s surface and limits the corrosion rate [5,6,7,8]. However, the contact of carbon steel with an acid–water mixture results in an unstable, hydrated deposit, which swells and does not provide a protective effect. The higher the water content in that mixture, the higher the corrosivity [5,6,7,8]. The corrosion process also takes a local form but occurs over a much larger area, posing a risk of perforation over the entire installation’s substations. In the case of carbon steels, the corrosion is accompanied by the hydrogen embrittlement phenomenon. Intense corrosion is caused by (apart from the hydrofluoric acid streams) gasoline containing hydrofluoric acid and water in emulsion form. The ultrasonic and coupon methods are effective in monitoring such corrosion risk.

At high temperatures, the most typical forms of corrosion are high-temperature sulphidic corrosion and naphthenic acid corrosion (NAC). Above 260 °C, hydrogen sulphide, which forms as a result of the thermal degradation of sulphur compounds contained in crude oil or due to the desulphurization process, is corrosive and usually causes a general attack on carbon steel (at low concentrations of naphthenic acids) [9]. A reaction between hydrogen sulphide and steel material is given below:

Fe + H_2_S = FeS + H_2_ (2)

The kinetics of this corrosion type has been thoroughly recognized and described by Gutzeit [10]. Temperature ranges of that corrosion mechanism and susceptibility of various materials have been investigated in detail [11,12]. High-temperature sulphidic corrosion is usually described as a general mass loss or degradation of exposed metal surfaces with the formation of sulphide deposits. In contrast, naphthenic acid corrosion has a more local character. Corrosion of carbon steel in naphthenic acids is a more complicated process due to the complexity of the naphthenic acid mixture [13,14,15,16]. Nevertheless, this type of corrosion can be generally presented using the following equation, when iron is the corroding material:

Fe + 2RCOOH = Fe(RCOO)_2_ + H_2_ (3)

Corrosion due to naphthenic acid is characterized as a local attack, especially in the zones of high flow rate of the medium or of significant condensation of concentrated acid—thus in the distillation columns and transmission lines. No corrosion products are observed on the material’s surface as they are soluble in crude oil, leaving exposed bare metal. Accordingly, NAC can reach unexpectedly high rates and cause local attacks.

Both aforementioned corrosion mechanisms can occur simultaneously in the petroleum industry and that is why they can be competitive. Naphthenic acids RCOOH react with iron, that being a construction material of the refinery equipment, producing iron naphthenates Fe(RCOO)_2_, which are soluble in the oil phase. Hydrogen sulphide dissolved in crude oil can react with iron forming iron (II) sulphide FeS, which is insoluble in the oil phase. Additionally, hydrogen sulphide (if present in high amounts) can react with formed iron naphthenates and recover naphthenic acids, which once again react with steel—in this way, the cycle is repeated and material degradation is intensified.

These processes take place in the installations of crude oil distillation, gasoline, diesel, and soft asphalt desulphurization and hydrocracking. Corrosion risk has been substantially minimized due to the implementation of low-alloy steels that are relatively resistant to this form of attack. Monitoring is accomplished mainly with electrical resistance, coupon, and ultrasonic techniques. Utilization of the electrochemical techniques is not possible because of a lack of aqueous phase.

In lower-temperature streams where water condensation occurs, corrosion in sour water is observed. The corrosion rate is primarily dependent on the pH of the aqueous environment. The form of the ionic medium is a decisive factor. Depending on pH, hydrogen sulphide in an aqueous environment can be present in the following forms: H_2_S unionized, HS^−^, and S^−2^ [17,18]. A decrease in pH is accompanied by a higher corrosion rate, which is usually connected with an increase in hydrogen sulphide content in water (assuming no impact from other chemical constituents). The second factor is temperature [19]. Generally, the corrosion rate increases with rising temperature due to an increase in the kinetics of the electrochemical reactions (on the other hand, solubility of hydrogen sulphide in water decreases). Other factors are stream flow rate and oxygen content [20]. It is believed that at above 50 ppb of oxygen in water the corrosion rate significantly increases. It must be emphasized that during the electrochemical corrosion reactions in a hydrogen sulphide environment, hydrogen evolves, initially in the atomic form and finally in the molecular form inside the steel structure. This phenomenon results in hydrogen embrittlement, also called wet H_2_S degradation [19]. Depending on the ammonia content in a stream (formed as a result of desulphurization or thermal degradation of organic compounds containing nitrogen), the pH of so-called sour water can be alkaline. Then, the degradation is termed corrosion in alkaline water and its rate depends mainly on ammonia salt content, temperature, and flow rate of the stream. A similar corrosion mechanism can be found in the amine washing installations. High amounts of hydrogen sulphide and carbon dioxide are dissolved in amines. Elevated temperature and high flow rates typically result in general corrosion, often in the zones with higher flow rates. The most effective method of corrosion monitoring of the above corrosion mechanisms is the linear polarization resistance one, although the electrical resistance and coupon methods are also utilized.

Many other corrosion mechanisms also occur in petroleum and petrochemical installations. A significant threat is atmospheric corrosion [21,22] of non-insulated components of the installation and the possibility of corrosion under the insulation [23,24] in the temperature range where water condensation may take place. There are also many corrosion mechanisms that affect smaller zones. For streams containing water in liquid form, the following can be mentioned: colling water corrosion [25,26], boiler water condensate corrosion [27,28], and CO_2_ corrosion [29,30]. In streams in which lye is used for neutralization and desulfurization, a caustic corrosion mechanism occurs [31,32]. In the range of high temperatures exceeding 204 °C, high-temperature corrosion processes such as oxidation [33,34], nitriding [35], and metal dusting [36,37,38] may proceed.

The aim of this paper is to present the main methods of corrosion monitoring in the petroleum industry from a practical standpoint, highlighting various aspects stemming from our long-term experience in this field. The presented results are meant to indicate the areas where special emphasis should be put on reliable corrosion monitoring.

## 2. Analysis of the Corrosion-Monitoring Methods in Petroleum Industry

A wide range of corrosion-monitoring methods are used in many branches of industry. In the petroleum and petrochemical industries, the number of measurement methods is limited. An important factor is the presence of explosive and flammable environments outside the installation, which significantly complicates the construction of electronic measurement systems. The second significant difficulty is access to monitoring points. Petroleum installations often have high columns and tanks, which generally calls for building completely autonomous, online systems. The most commonly used corrosion-monitoring methods are discussed below: NDT, ER, LPR, and the coupon method.

### 2.1. Non-Destructive Testing Methods in Corrosion Monitoring

The application of non-destructive testing (NDT) methods to corrosion monitoring has become one of the most extensively developed fields of corrosion rate measurement during the last couple of years. Non-invasive measurement is the main advantage of the NDT methods. In the case of classic corrosion methods, it is necessary to provide holes in the installations to fit the sensors. Such a solution is complicated due to logistic aspects. A corrosion-monitoring system can be installed exclusively during the repair shut-down periods, which take place at several-year intervals, depending on the type of installation. The costs related to sensor mounting are high as they are connected not only with purely mechanical operations but also with the preparation of necessary documentation and execution of the investigations indispensable for safety certificate issuing (because of the temperature and pressure conditions). All mounting operations must be correlated with other repair and inspection procedures during the shut-down period, which is not an easy task. The cost of lock/bypass for the corrosion sensors significantly increases for the installations with high temperature and pressure parameters. Mounting of the corrosion-monitoring sensors imposes an additional risk of failure due to unsealing upon long-term service. Another advantage of utilization of the NDT methods for corrosion monitoring is the fact that an actual corrosive system, not the sensor simulating the corrosion process, is subjected to monitoring. Monitoring takes place on the part of the installation exposed to actual operating conditions. Accordingly, all the requirements for reliable measurement are fulfilled: suitable exposure time (formation of deposits and corrosion products in a natural way), and no perturbations in the technological medium flow (there is no sensor, which can disturb the flow of the medium). The next advantage of NDT is the possibility of monitoring any parts of the installation, especially where mounting the invasive sensors is impossible, for instance, due to limited access or extremely severe temperature and pressure conditions. Selection of a monitoring site can be done practically at any time, without the necessity of the installation shut-down. Moreover, it is possible to perform monitoring in a few points close to each other, which allows reliable analysis of local corrosion kinetics as well as erosion damage. Currently, there are two NDT corrosion-monitoring methods available:

- Profile radiographic testing. This method utilizes a typical radiographic method that is usually employed in a wall thickness inspection. An important advantage of this solution is the possibility of measurement execution without the removal of the insulation as well as in hardly accessible places without the need for direct contact with the examined construction element.
- Ultrasonic thickness measurement. This method has been used for years; however, the progress in measurement systems allows for online testing. That is why this method can be classified as a corrosion-monitoring system.

**Figure 2:**
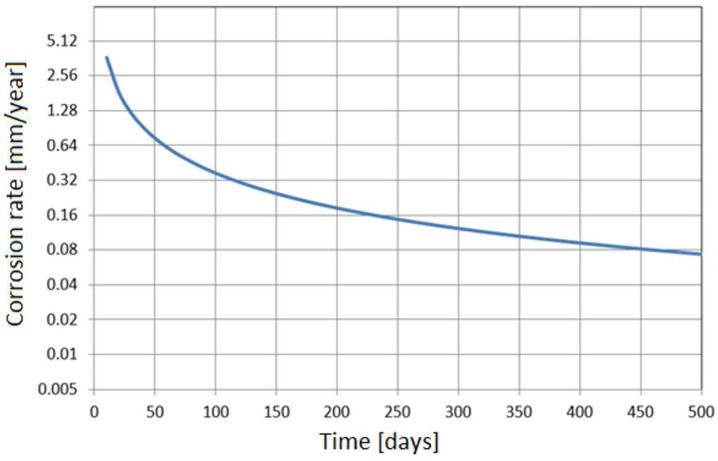
Simulation results of corrosion rate measurement using the UTM method versus time of measurement.

An important feature of each corrosion-monitoring system is the accuracy of the measurement. In the case of the NDT methods, corrosion loss can be unequivocally determined. The minimum resolution of the ultrasonic wall thickness measurement is 0.01 mm. Figure 2 shows the results of the accuracy of corrosion rate measurement versus the time of measurement.

**Figure 3:**
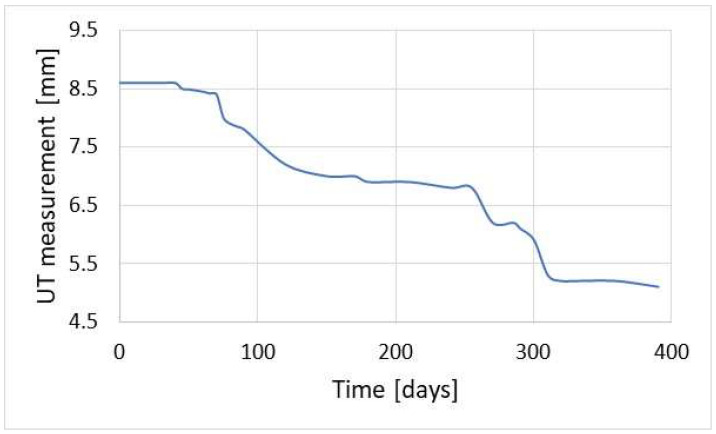
Corrosion loss obtained with the monitoring system based on ultrasonic measurement of pipeline wall thickness.

The results presented in Figure 2 should be interpreted as the accuracy of corrosion rate measurement. The figure provides the minimum time, for which the corrosion rate can be determined. For high rates, due to a substantial corrosion loss, the results can be obtained after a short time. Figure 3 illustrates corrosion loss determined with the ultrasonic monitoring system. The presented results show that the UTM method is very useful as it provides precise information about the wall thickness of a particular construction element.

**Figure 4:**
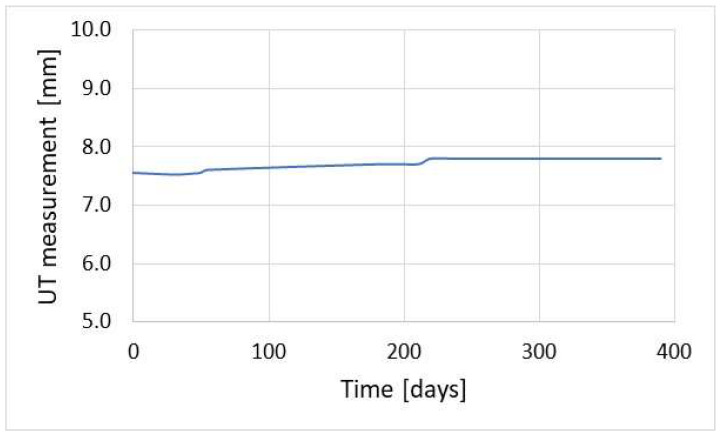
Exemplary measurement of pipeline wall thickness with online UT method upon the presence of well-adherent deposit (FeF_2_).

In the case of low corrosion rates, there is a small corrosion loss; thus, reliable results of the corrosion rate are achieved after much longer time, which is inconvenient from a practical standpoint. For instance, a typical threshold value of corrosion rate of 0.125 mm/year (the threshold between medium and high corrosion rate according to the NACE SP0775-2013 standard) is measurable after about 28 days [39] (Figure 2). This level of corrosion risk cannot be measured in a shorter time, so during continuous corrosion monitoring reliable results indicating the corrosion rate of 0.125 mm/year are available after ca. 28-day time span. Another difficulty during thickness measurements is the formation of deposits. When tight and highly adhesive deposits are present, wall thickness measurement can be burdened with an error. In practice, this problem occurs very seldom; however, one has to be aware of such potential measurement complications. Figure 4 presents the results of online UTM upon the conditions of FeF_2_ deposit formation.

Figure 4 shows an increase in pipeline wall thickness. In this location, there was simultaneous corrosion monitoring using the coupon method, the results of which are gathered in Table 1. Monitoring using the coupon method was performed every 60 days. The last measurement cycle was longer and lasted for 120 days.

**Figure 5:**
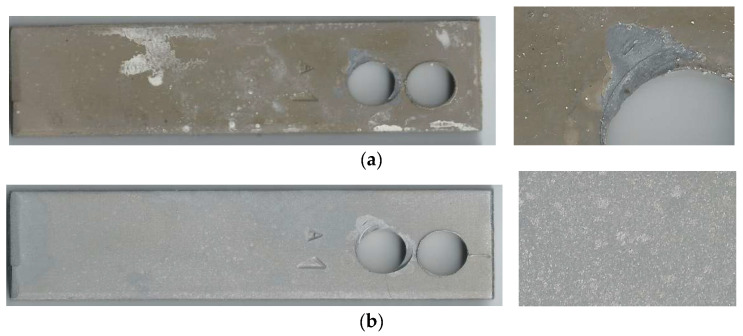
Condition of the coupon exposed to a technological stream containing hydrofluoric acid: (**a**) after exposure; (**b**) after cleaning.

The corrosion rate results presented in Table 1 are in contradiction with the results illustrated in Figure 4. This can be explained by formation of well-adherent deposits (mainly FeF_2_). The condition of the coupon’s surface before and after cleaning is shown in Figure 5. Data analysis confirmed the causes of measurement errors during pipeline wall thickness measurement using the UT method.

Many laboratory techniques are employed to obtain valuable information regarding the explanation of corrosion mechanisms. These include morphological tests [40,41] and metallographic investigations.

### 2.2. Electrical Resistance Method

**Figure 6:**
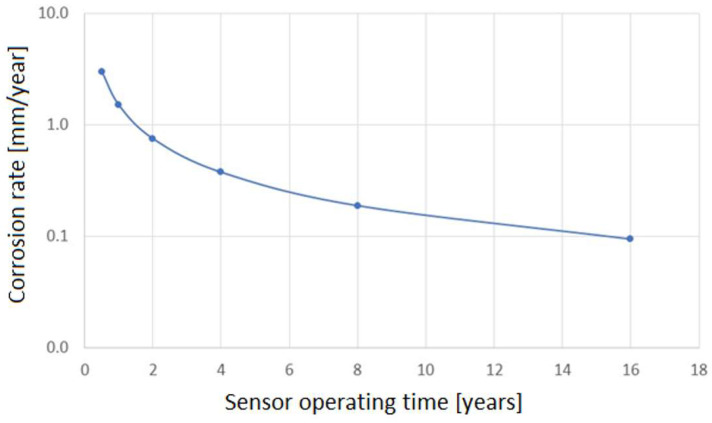
Dependence between corrosion rate and operating time of the ER sensor, calculated for sensor’s measurement element thickness—4 mm.

The electrical resistance method is one of the most popular online monitoring techniques due to the lack of limitations connected with the type of corrosion environment where the measurement is carried out. One of its main advantages is the possibility of determining an average corrosion rate. The obtained corrosion rate value relates to the period between successive measurements, which allows assessment of the lifetime of a given installation. Generally, the longer the operation time of a sensor, the higher the accuracy of measurement because of the increasing accuracy of raising electrical resistance. Unfortunately, the limitations are a risk of sensor damage due to corrosion and a possibility of local corrosion of the sensor, which results in measurement errors. Figure 6 illustrates a dependence between corrosion rate and the ER sensor operating time.

**Figure 7:**
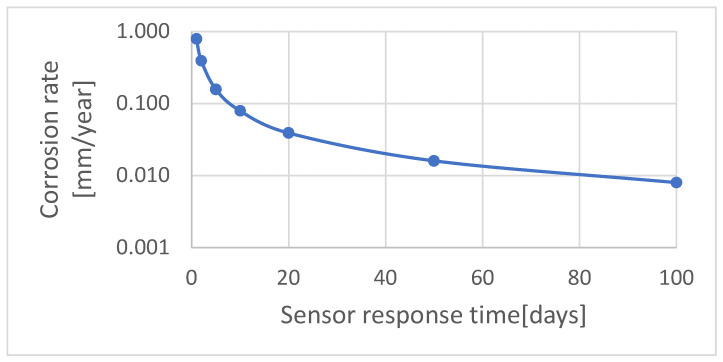
Dependence between expected corrosion rate and time of correct measurement data acquisition, calculated for sensor’s measurement element thickness—4 mm.

Figure 6 shows that for corrosion rates higher than 5 mm/year, safe operation of the sensor does not exceed one year. When the corrosion rate is lower than 0.1 mm/year, the sensor is durable for several years. Commercial ER sensors are produced with a certain range of measurement element thickness. Higher thickness contributes to a longer lifetime (thicker elements can be subjected to higher corrosion losses). However, in this case, the sensor’s resistance drops, which results in a decrease in measurement accuracy. Generally, thicker sensors are designated for relatively high corrosion rate measurement but their accuracy is insufficient at low corrosion rates. The sensors with low thickness of the measurement element are characterized by higher sensitivity in the range of low corrosion rates. Figure 7 depicts a dependency between the expected corrosion rate and the time of correct measurement data acquisition. It shows that at low corrosion rates, below 0.01 mm/year, reliable information about the corrosion process can be obtained after a few months of exposure.

**Figure 8:**
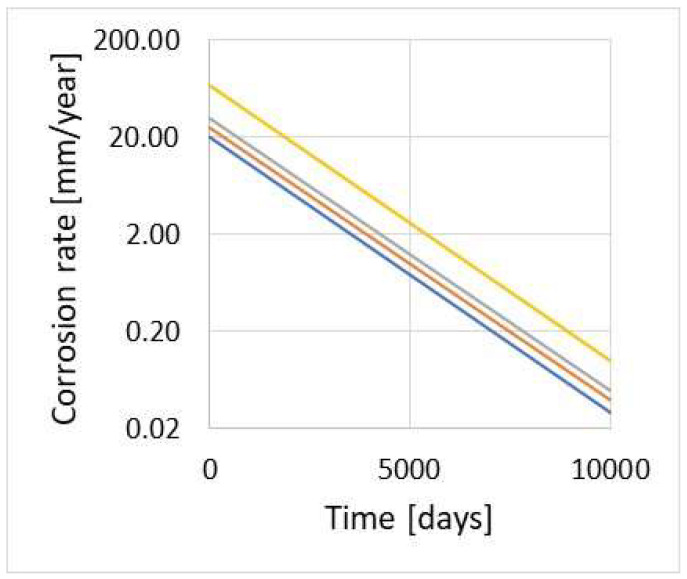
Example plot for selection of the ER sensor; thickness of measurement element: yellow 1 mm, grey 0.25 mm, orange 0.12 mm, blue 0.1 mm.

Producers of the sensors recommend a selection of the sensor’s parameters (measurement element thickness) for the expected corrosion rate. Such an approach contributes to higher accuracy of measurements and limitation of the risk of premature sensor damage due to corrosion. Figure 8 presents an example plot facilitating the selection of the ER sensor for the expected corrosion rate and time of measurement data acquisition.

**Figure 9:**
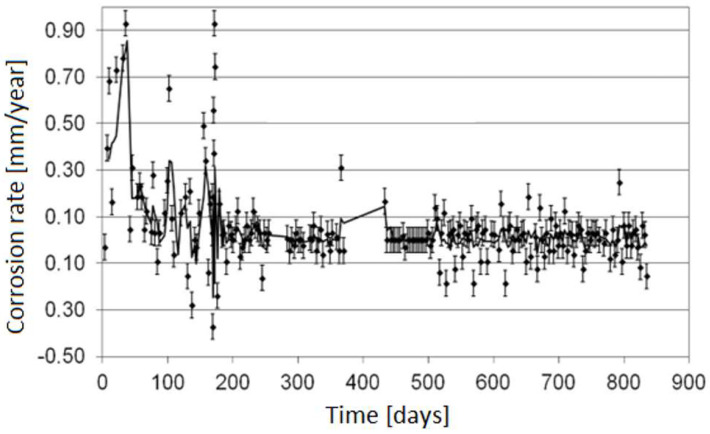
Results of corrosion monitoring in a stream of hydrocarbons using the sensor with 0.9 mm thick measurement element upon the conditions of varying corrosion rate (periodical increase in the corrosion rate).

Unfortunately, it is not possible to produce a fully universal sensor characterized by high accuracy in the range of low- and high-corrosion rates at the same time and a long lifetime. Figure 9 depicts the results of corrosion monitoring conducted in a stream of hydrocarbons using the sensor with a 0.9 mm thick measurement element upon the conditions of periodical increase in corrosion rate beyond 1 mm/year.

**Figure 10:**
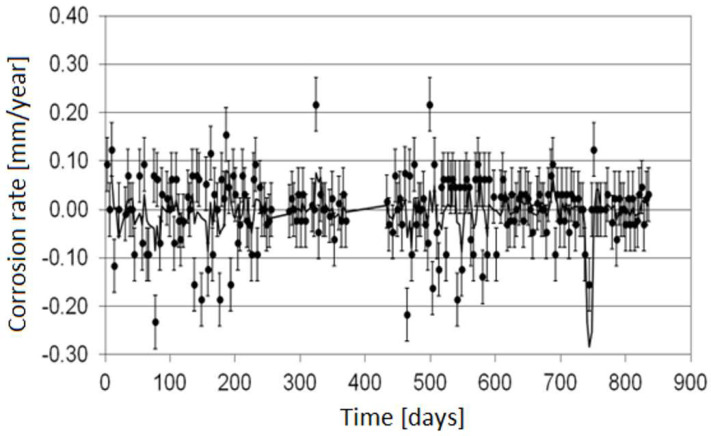
Corrosion rate results obtained using the sensor with a 0.9 mm thick measurement element upon correct operation of the installation (no significant increase in the corrosion rate).

The results prove that the online ER measurements provided fast detection of corrosion risk, which allowed a change in process parameters, resulting in a decrease in corrosion aggressiveness of the stream. Figure 10 depicts the corrosion rate results obtained with the same sensor during the correct operation of the installation (no significant increase in the corrosion rate).

**Figure 11:**
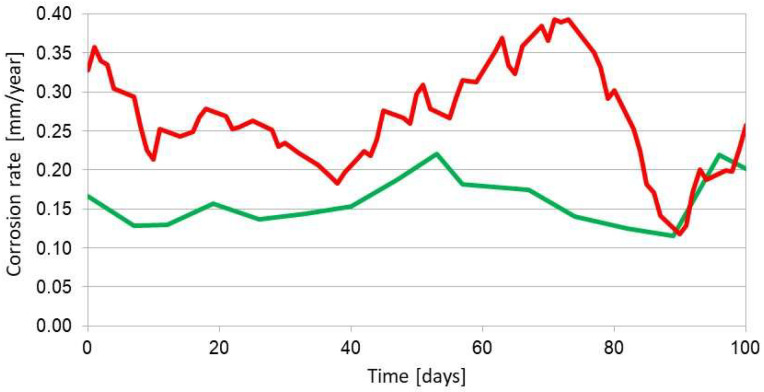
Example of corrosion monitoring with the ER method (red curve) upon the presence of corrosion and degradation phenomena. The green curve represents the results of the coupon method. Material: A109 gr. B steel, environment: water saturated with H_2_S.

**Figure 12:**
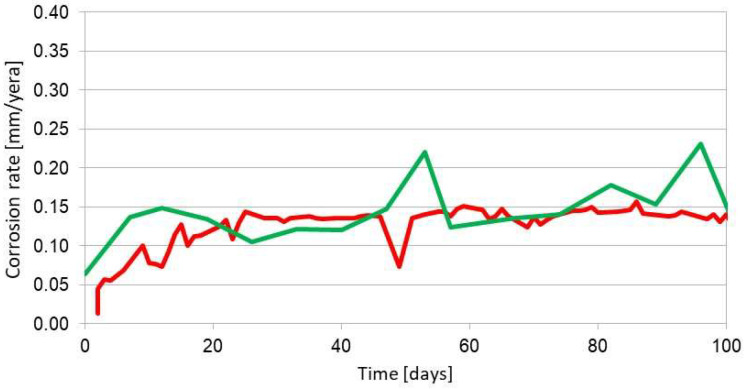
Example of corrosion monitoring with the ER method (red curve) upon the presence of corrosion phenomena only. The green curve represents the results of the coupon method. Material: A109 gr. B steel, environment: 3.5% aqueous NaCl solution.

The results of measurements from more than two years of exposure do not indicate the occurrence of corrosion processes. A comparative measurement with the coupon method revealed a corrosion rate at the level of 0.003 mm/year. Such a low corrosion rate is almost undetectable with this ER sensor. There are solutions, which partially solve the problem of poor universality of the ER method. Two measurement elements with different thicknesses were accommodated in a single sensor [42]. Unfortunately, at high corrosion rates, the element with lower thickness can be easily damaged. The idea of the ER measurements is an analysis of corrosion loss of the measurement element, so the ER sensors are disposable and cannot be regenerated when the measurement element is consumed by corrosion. The ER measurement may not be feasible in environments with high conductivity, for instance, in molten metals and salts. Then, electric current is not going to flow through the entire length of the measurement element but cover the shortest possible path, making correct measurement impossible. Short, rapid changes in ambient temperature also make reliable measurements impossible. Such conditions occur inside the distillation columns. The value of resistance is difficult to evaluate as the reference element in the ER sensor is characterized by much higher temperature inertia, which can result in an error during resistance measurement upon fast temperature changes of the corroding element in the sensor. Corrosion monitoring with the ER method can yield an overestimated corrosion rate if apart from the corrosion processes there are also other degradation phenomena (cracking, structural changes). Such a situation is illustrated in Figure 11. Figure 12 shows the results of analogous corrosion monitoring but in an environment where only corrosion processes take place.

**Figure 13:**
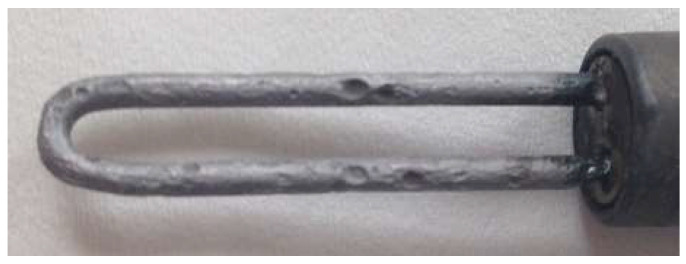
Local corrosion of the ER sensor during exposure to a liquid gas environment.

Degradation processes (structural changes, cracking) contribute to an increase in resistance, which results in an overestimation of the corrosion rate, which is clear in Figure 11. When only corrosion processes occur, the ER method yields correct corrosion rate values. Errors in the estimation of the corrosion rate can appear also when local corrosion of the measurement element takes place. It must be emphasized that the ER method allows the detection of local corrosion because there is a significant increase in sensor resistance attributed to local depletion of sensor thickness (Figure 13). The calculated corrosion rate is not going to agree with the actual value as the calculation mathematical model accounts for general corrosion.

### 2.3. Monitoring with Linear Polarization Resistance Method

**Figure 14:**
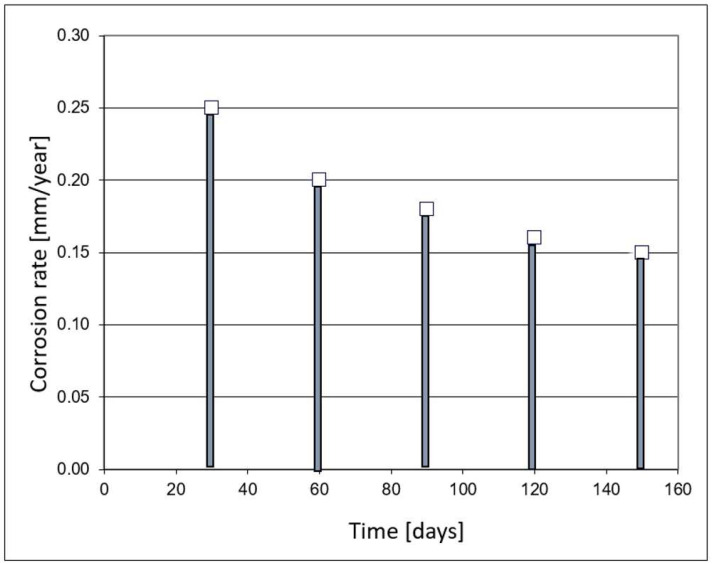
Example result of corrosion rate measurement using the LPR method.

Corrosion monitoring with the linear polarization resistance method can be performed only in electrolytic environments (mainly aqueous systems). The corrosion rate determined with LPR is instantaneous in character. Figure 14 presents the idea of instantaneous corrosion rate measurement.

**Figure 15:**
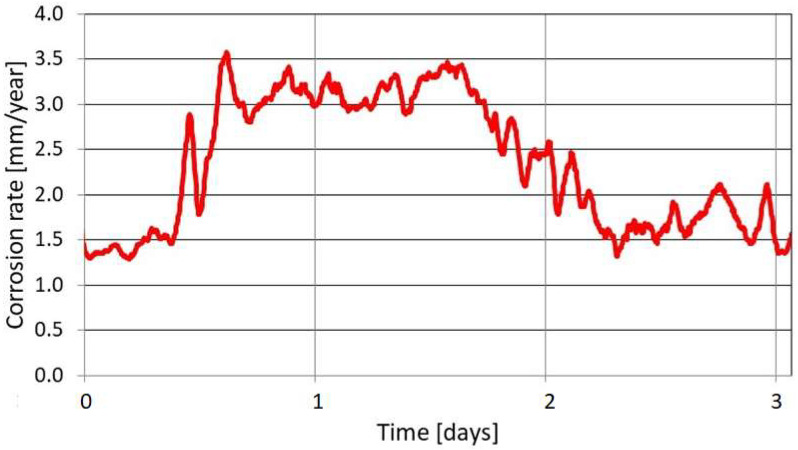
Results of corrosion monitoring with the LPR method. Changes in corrosion rate value originate from instantaneous changes in the chemical composition of the aqueous environment. Corrosion in water with high CO_2_ content.

In the case presented in Figure 14, the corrosion rate measurement is executed at 30-day intervals. A particular result corresponds to an exact time instant of measurement act; thus, the obtained value is instantaneous. Accordingly, the condition of a corroding system is unknown between successive measurements. The non-destructive character of LPR makes it possible to increase the frequency of the measurements (for instance every 3 h), so this approach is often employed in online corrosion monitoring. In this case, corrosion rate monitoring reflects actual corrosion risk conditions. For instance, this method allows monitoring of corrosion rate changes due to variations in flow rate or environment composition. Figure 15 shows the results of corrosion monitoring in an aqueous environment, the chemical composition of which changes during the measurement.

**Figure 16:**
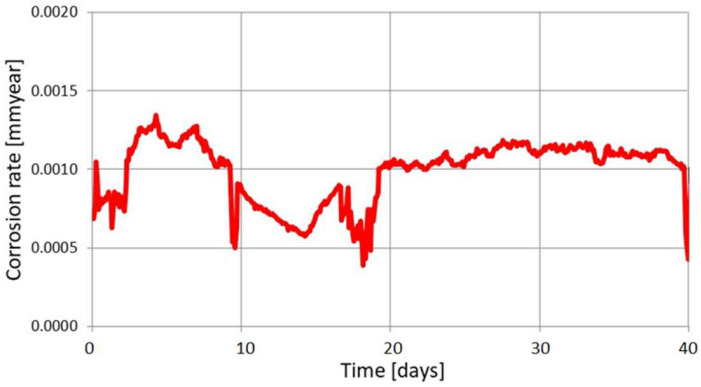
Results of corrosion monitoring with the LPR method, measurement in water with low oxygen content.

The LPR measurement also allows the detection of very low corrosion rates. Figure 16 illustrates the results of corrosion monitoring in an aqueous system with very low oxygen content (10 ppb) and a temperature of 15 °C.

**Figure 17:**
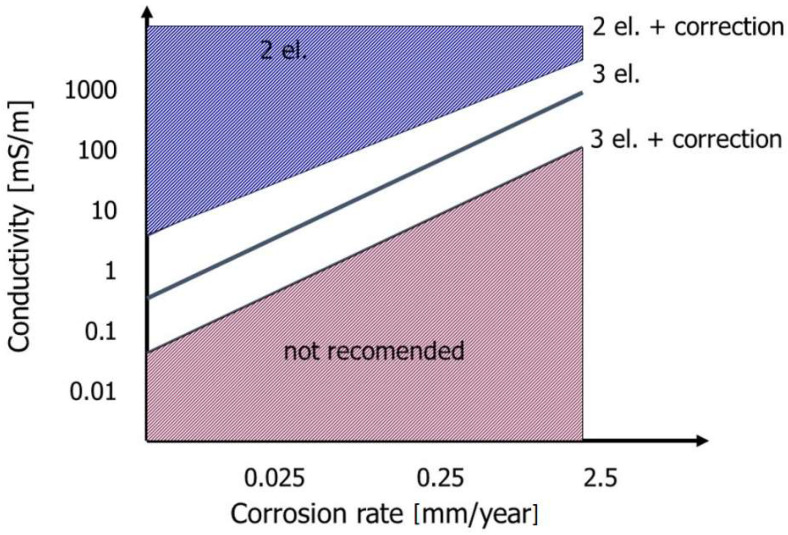
Plot illustrating the methodology of measurement error limitation due to electrolyte resistance where 2 el.—2-electrode sensor; 3 el.—3-electrode sensor.

Upon corrosion monitoring in the aqueous solutions with low conductivity (demineralized water, rainwater), the results are influenced by electrolyte resistance. The electrolyte resistance and charge transfer resistance cause voltage (potential) drop during the measurement. The electrolyte resistance is connected with the conductivity of the investigated solution and depends on the number of soluble chemical compounds, which undergo dissociation. Dissociation produces ions, which are responsible for the conduction of electric current in aqueous environment. The charge transfer resistance describes the properties of electrochemical processes connected with the recombination of ions on the electrode’s surface. The value of this resistance is closely related to the corrosion rate, which is a fundamental indicator of corrosion aggressiveness of the environment. The LPR measurement allows the determination of the polarization resistance, which is a sum of electrolyte resistance and charge transfer resistance: R_P_ = R_E_ + R_ct_. The value of the electrolyte resistance can be acquired from other measurements, for instance, conductivity or impedance measurements. Producers of the LPR corrosion-monitoring systems provide the plots facilitating the selection of a sensor depending on the conductivity of a medium and expected corrosion rate. An example plot for the selection of the sensors for the given electrolyte conductivity (corrosion environment) is shown in Figure 17.

**Figure 18:**
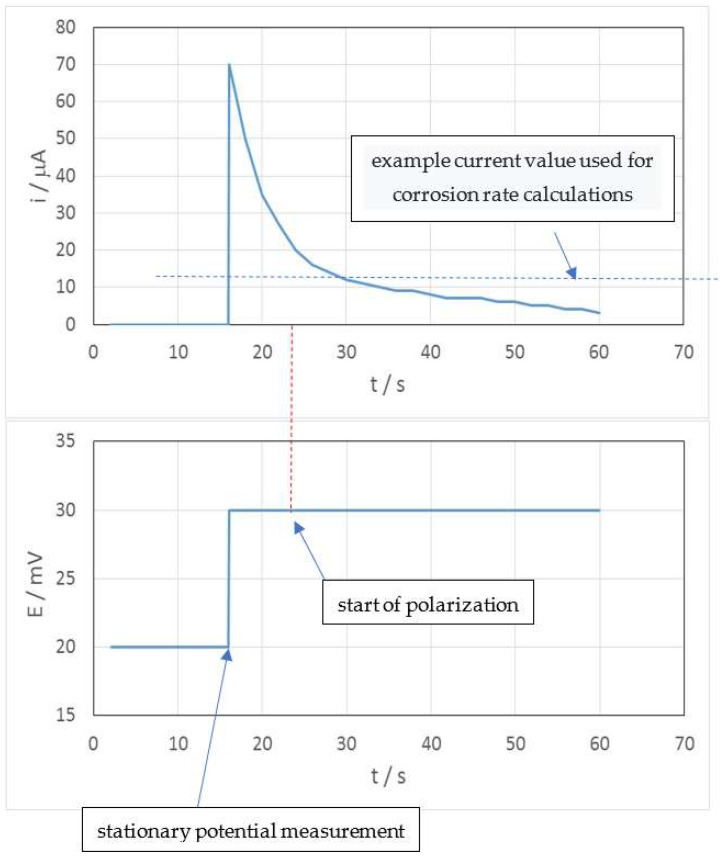
Example potential and current records from a single-point LPR measurement.

Generally, different sensors (2- or 3-electrode ones) and correction results for estimated electrolyte resistance are used depending on the resistance of the medium. The LPR results are burdened with a so-called chronovoltamperometric effect error, which hinders the determination of the correct value of current. Figure 18 presents the example potential and current records from a single-point measurement.

A significant drawback of the LPR measurement is a problem with the determination of the value of the current flowing in the measurement system during polarization. The current decreases during the measurement. The value of the current is usually determined as an average over the entire measurement or as a fixed value corresponding to a given moment of measurement. Changes in current value result from the polarization of the electrode. Partial elimination of the error is achieved via the execution of the measurement in galvanostatic conditions. This solution fails for big variations in the corrosion rate because there is a high probability of non-linear system behaviour when a voltage signal significantly exceeds 10 mV. This kind of error was analyzed in the literature [43,44,45]. It was found that the linear range depended on the value of Tafel coefficients (resulting from the Butler–Volmer reaction). When the Tafel coefficients are low, the linear range can be only ±2 mV. For high Tafel coefficients, the linear range extends to ±60 mV. The linear range also depends on the corrosion current value. The linearity of the system is limited for low values of polarization resistance. The problem of linearity is also connected with control over the corrosion process, which is described by the Tafel coefficients. In the case of significant differences between $\beta_{a}$ and $\beta_{c}$, the linear ranges in anodic and cathodic directions are different.

The LPR measurement is sensitive to the errors originating from the instability of the measurement system. The LPR monitoring requires stationarity conditions in the system under investigation (similar to the EIS measurements). If the stationary potential changes during the measurement, the current value is determined with a statistical error. Corrosion rate results are under- or overestimated in such situations, depending on the direction of the stationary potential change. The errors most frequently originate from:

- Oscillation of potential due to the presence of active-passive cells;
- Changes of potential caused by variations of environmental conditions (flow, temperature, oxygenation).

**Figure 19:**
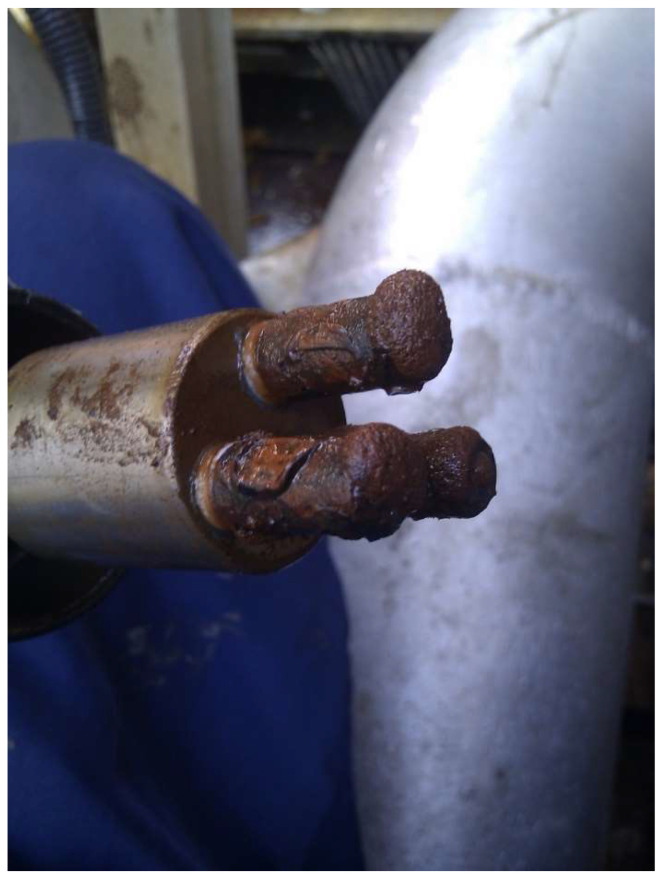
Condition of a sensor after 90 days of exposure to brine.

Commercial measurement systems offer the possibility of evaluation of changes in stationary potential before the LRP test. In the case of oscillations or variations of potential, the measurement procedure can be extended over time to arrive at the required potential stability. Corrosion monitoring with the LPR method can generate an error connected with the accumulation of corrosion products on the electrode’s surface [43]. In automated monitoring systems, sensor exposure time is usually very long. In particular environmental conditions, large amounts of insoluble, relatively well adhering corrosion products form on the electrode’s surface. This results in a significant change in the surface area of the working electrode, causing substantial error in corrosion rate determination. Figure 19 shows the condition of a sensor after 3 months of exposure to brine.

The results of the corrosion rate obtained with the LPR method can differ with respect to the ones from the coupon method. The reason is the fact that the coupon tests provide an average corrosion rate, while LPR provides an instantaneous corrosion rate [46].

A much more advanced technique for monitoring corrosion is the electrochemical impedance spectroscopy (EIS) method [47,48,49]. This measurement method employs an alternating current sinusoidal signal. The measurement, as in the case of LPR, is performed within the linearity range of the electrochemical system. An important advantage of the method is the ability to determine the resistance of the electrochemical environment during measurement, which allows for a much more accurate determination of the charge transfer resistance, which is proportional to the corrosion rate. The practical application of this method in corrosion monitoring is limited to corrosion in acidic environments. The reason is the fact that in the case of diffusion limitations, there are significant difficulties in determining the parameters of the electrical equivalent circuit automatically. It is also possible to monitor corrosion with localized techniques such as scanning vibrating electrode technique (SVET), localized electrochemical impedance spectroscopy (LEIS), scanning electrochemical microscopy (SECM), scanning kelvin probe (SKP), scanning ion-selective electrode technique (SIET), and scanning droplet cell (SDC) technique [50]. However, due to the high level of complexity of measurement execution, their use is currently limited mainly to laboratory conditions.

### 2.4. Monitoring with Coupon Method

**Figure 20:**
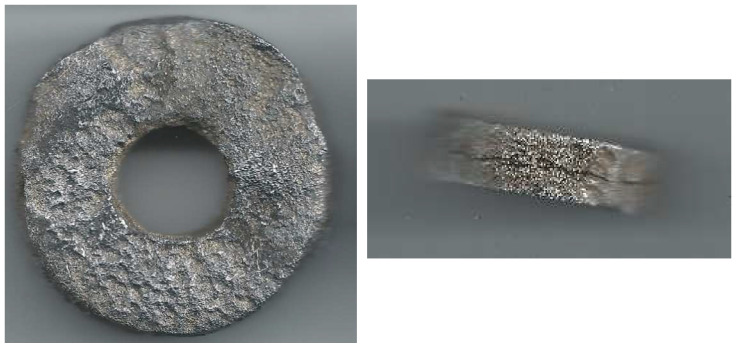
Coupon after 420 days of exposure to aqueous environment saturated with H_2_S.

**Figure 21:**
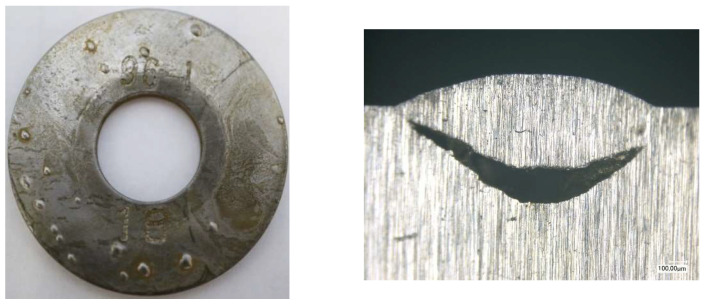
Coupon after 330 days of exposure to vapors from second-stage hydrodesulfurization (HDS) reaction.

The coupon method is one of the cheapest and the oldest corrosion-monitoring techniques. This method, when combined with the microscopic and NDT techniques, allows determination of the corrosion mechanism. Figure 20 illustrates the surface of a coupon, which apart from corrosion loss exhibits also a cracking mechanism (aqueous H_2_S environment). Figure 21 presents the coupon suffering from hydrogen-induced cracking/stress-oriented hydrogen-induced cracking (HIC/SOHIC), upon condensation of moisture with a significant amount of H2S.

**Figure 22:**
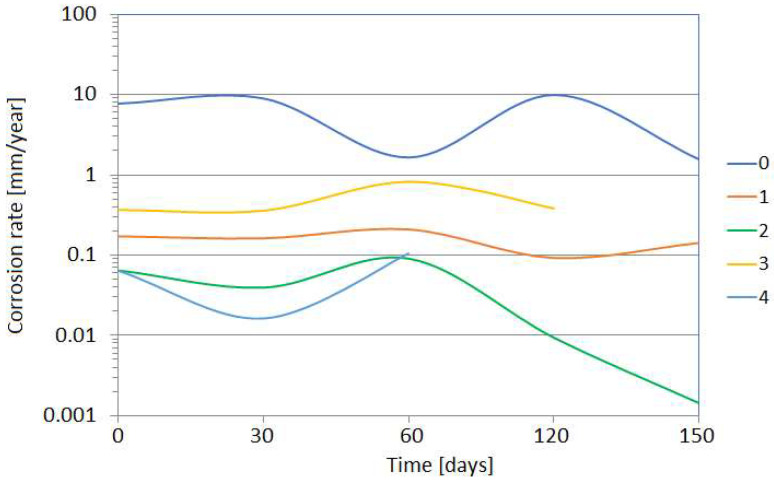
Results of corrosion rate of the coupons exposed in the alkylation unit to corrosion environments of different aggressiveness for 2 months: 0—alkylate with hydrofluoric acid and water; 1—n-butane with traces of hydrofluoric acid and water; 2—hydrofluoric acid (after water removal); 3—isobutane with hydrofluoric acid and water; 4—polymer with traces of hydrofluoric acid and water.

**Figure 23:**
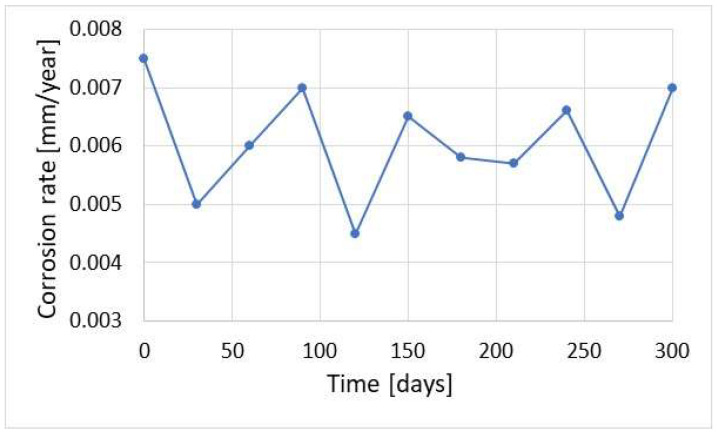
Results of corrosion rate obtained with the coupon method during 2-month exposure in mixed hydrocarbon/water environment.

Corrosion monitoring with the coupon method is universal and allows the determination of different magnitudes of corrosion rate with high accuracy. Figure 22 shows the results of the corrosion rate of the coupons exposed to different corrosive environments in the alkylation unit for 2 months. The measurement allowed evaluation of corrosion rates of 0.005 and 10 mm/year occurring inside the same technological installation. The results of the corrosion rate obtained with coupons strongly depend on their exposure time. Some variations can be attributed to both corrosion and sediment formation processes. The results of the corrosion rate in the sour water stream are shown in Figure 23.

**Figure 24:**
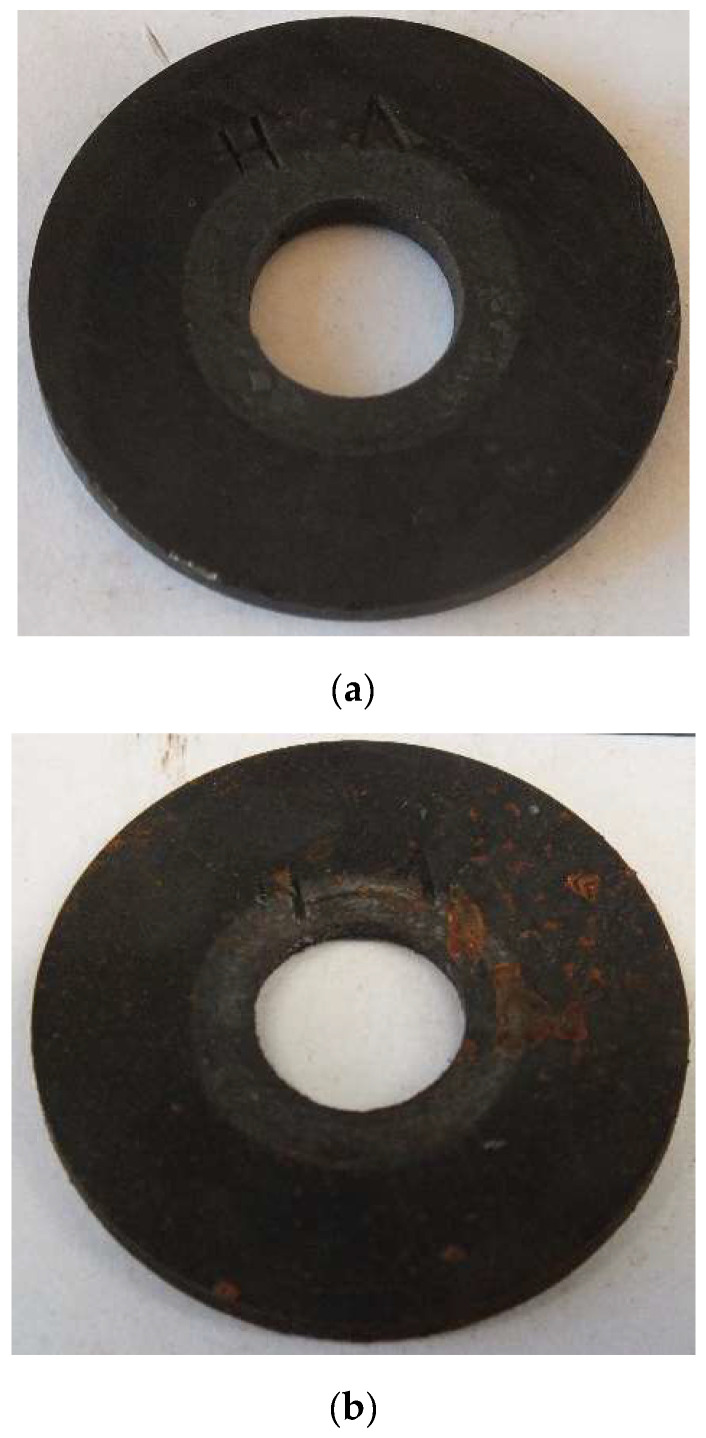
Condition of the coupons after (**a**) 60-day and (**b**) 180-day exposure to gasoline with traces of water.

The investigations were conducted in 2-month cycles and the entire coupon exposure period amounted to 162 days. The condition of the coupons after 2-month and 6-month exposure to gasoline with traces of water is illustrated in Figure 24. The corrosion rate determined for the long-term coupon was 0.0013 mm/year. The corrosion rate calculated as an arithmetic mean of the 2-month exposure was equal to 0.0059 mm/year, which was over 4 times higher than the corrosion rate of the long-term coupon.

**Figure 25:**
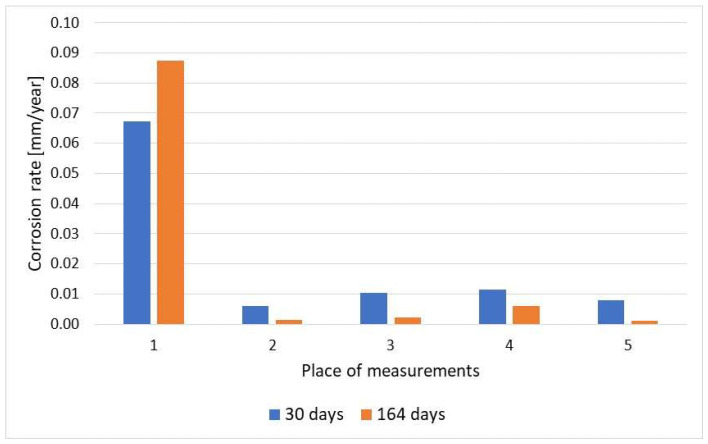
Comparison of corrosion rate results for the coupons exposed for 30 and 164 days.

**Figure 26:**
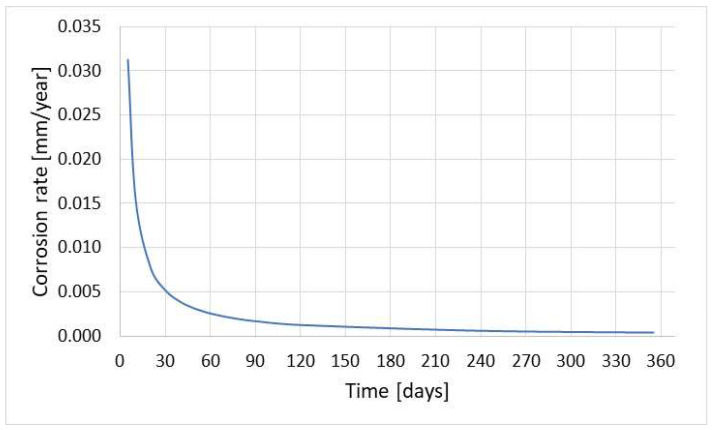
An error of corrosion rate measurement connected with self-corrosion of the coupon during cleaning.

Figure 25 illustrates the results of corrosion monitoring for various coupon exposure times in the hydrocarbon streams. Generally, in most cases, the corrosion rate determined from long-term coupon exposure is lower. The monitoring with the coupon method should take into account the error connected with the cleaning of the coupon. Removal of corrosion products from the coupon is conducted in hydrochloric acid with the addition of an acid etching inhibitor. Figure 26 presents the error of corrosion rate measurement related to coupon corrosion during cleaning. The data presented in Figure 26 concern the coupon of mass 11.6 g and surface area 30 cm^2^. It is evident that longer exposure time is associated with lower measurement error.

A very difficult aspect of corrosion monitoring is the components of an installation equipped with cladding. Due to a crevice between the substrate material and the cladding, it is impossible to measure both of these construction elements with the NDT methods. Corrosion monitoring is feasible only by implementation of the ER or LPR method where a working element of the sensor is made of a material identical to that of the cladding. Relating the obtained results to actual conditions could be troublesome.

An undisputable advantage of the coupon method is the possibility of direct observation of the exposed material and acquisition of information about the amount and type of sediments forming in the process stream.

Important data providing knowledge on the corrosion mechanisms and corrosion rates in refinery installations can be obtained based on accelerated tests conducted in the laboratory. In the case of atmospheric corrosion risk, the tests are carried out in climatic and salt spray chambers. Accelerated tests of organic coatings can be performed in UV chambers. For typical corrosion mechanisms in refineries, tests can be performed at elevated temperatures and for higher concentrations of corrosive agents.

## 3. Conclusions

Presented results obtained via various corrosion-monitoring methods allow the highlighting of different practical measurement aspects. The variety of utilized monitoring methods allows for obtaining a lot of valuable information regarding the kinetics of corrosion processes and the occurring corrosion mechanisms. The ultrasonic and ER methods are the most frequently used measurement methods that enable diagnosing the target lifetime of the installation. A significant disadvantage of these methods is the difficulty in measuring medium- and low-corrosion rates. Despite significant technical difficulties in execution, the coupon method is widely used because it allows the determination of the corrosion mechanism and, due to precise mass measurement, allows the determination of relatively low corrosion rates. The LPR method is generally underestimated in the petroleum industry because the aqueous phase frequently forming in refinery streams does not constitute a uniform layer, allowing correct measurements to be conducted. It is advantageous to use this method in sour water streams because the variation in their content of corrosive components, such as vapors, may change the corrosion rate. In this way, instantaneous modelling of the kinetics of highly corrosive streams can be performed. In general, all corrosion-monitoring methods require a thorough knowledge of the system under investigation, not only from a corrosion kinetics standpoint but also regarding technological aspects of the process. Corrosion-monitoring methods possess high potential for serious failure prevention; however, analysis of obtained data must be careful to avoid erroneous conclusions. Current development trends in corrosion-monitoring methods are clearly related to a significant expansion of the ultrasonic online method; however, certain limitations of this method should be remembered. The radiographic method offers significant measurement possibilities regarding pipeline wall thickness evaluation; in this case, it will be difficult to use this technique online. In recent years, more and more information has appeared on the application of the field signature method (FSM) [51] to corrosion monitoring in the petroleum industry. This measurement method is highly suitable for analysing the kinetics of erosion processes. It is mainly used in monitoring the elbow zones of critical pipelines.

## Figures and Tables

**Table 1:** Results of corrosion rate in hydrofluoric acid environment obtained with the coupon method.

No. of the Coupon Exposure Cycle	Corrosion Rate (mm/year)
1	0.171
2	0.162
3	0.209
4	0.092
5	0.142
Average	0.155

## Data Availability

The data presented in this study are available on request from the corresponding author. The data are not publicly available due to ongoing further studies in the field.
